# Zirconia-based catalyst for the one-pot synthesis of coumarin through Pechmann reaction

**DOI:** 10.1186/s11671-016-1525-3

**Published:** 2016-07-26

**Authors:** Shahid Ali Khan, Sher Bahadar Khan, Abdullah M. Asiri, Ikram Ahmad

**Affiliations:** 1 Chemistry Department, Faculty of Science, King Abdulaziz University, Jeddah, 21589 Saudi Arabia; 2 Center of Excellence for Advanced Materials Research (CEAMR), King Abdulaziz University, Jeddah, Saudi Arabia

**Keywords:** Zirconia, Heterogeneous catalyst, Coumarin, Pechmann reaction, Kinetic study, Room temperature, Solvent-free condition

## Abstract

Coumarins play an important role in drug development with diverse biological applications. Herein, we present the synthesis of coumarin through Pechmann reaction by using zirconia-based heterogeneous catalysts (ZrO_2_-TiO_2_, ZrO_2_-ZnO, and ZrO_2_/cellulose) in a solvent-free condition at room temperature. ZrO_2_-TiO_2_, ZrO_2_-ZnO, and ZrO_2_/cellulose were identified through spectroscopic techniques such as FESEM, X-ray, EDS, XPS, and FT-IR. ZrO_2_-TiO_2_ showed the best catalytic performance while ZrO_2_/cellulose was inactive. The kinetic parameters were observed in a solvent-free condition as well as in toluene and ethanol. The temperature effect was extensively studied which revealed that increasing the temperature will increase the rate of reaction. The rate of reaction in a solvent-free condition, ethanol, and toluene were 1.7 × 10^−3^, 1.7 × 10^−2^, and 5.6 × 10^−3^ g mol^−1^ min^−1^, respectively.

## Background

**Fig. 1:**
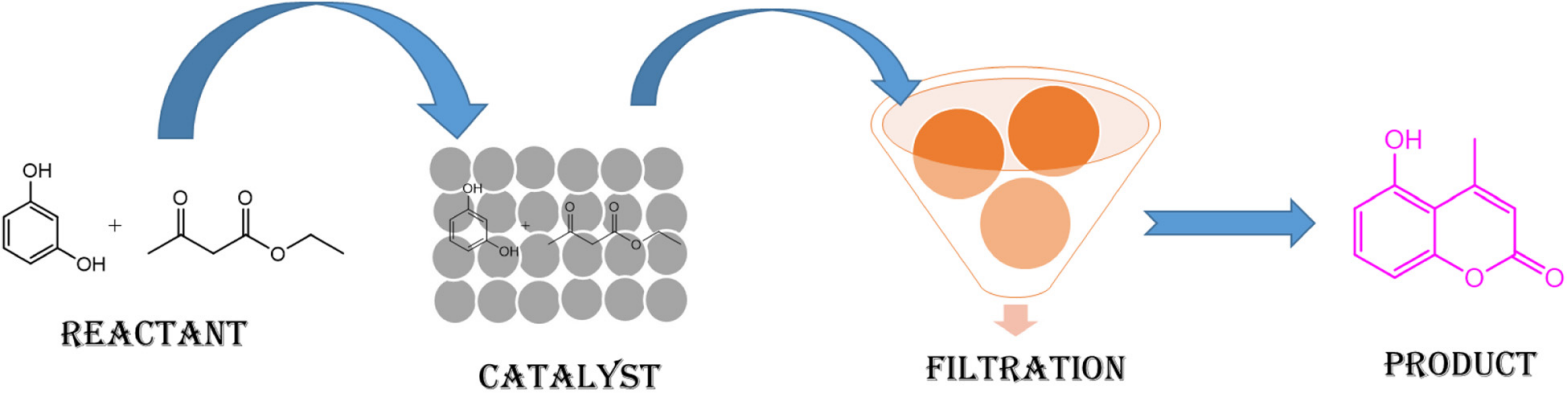
Diagrammatic view of the reaction

Heterogeneous catalysts play an extremely important role in the chemical industry which shows its applicability in our daily life [1]. Recently, scientists greatly reverted their attention towards the application of heterogeneous catalyst in the synthesis of important pharmaceutical scaffolds. It was estimated that more than 90 % of the chemical manufacturing depends on the catalytic processes [1]. The design and development of a catalyst with unique morphological and structural characteristics are the main focus in the field of catalysis [2]. The catalytic performance of a catalyst largely depends on the structural features and chemical composition, which in turn affect the active site of the catalyst, approachability of the molecules to the pore size of the catalyst and reactant product mass transport of the molecules [3–7]. A number of transition and normal element metal oxides (s and p blocks element) was largely used in various fields. Among transition metal oxide, zirconia (ZrO_2_) played an important role as heterogeneous catalyst, due to its dual nature (both acidic and basic) and semiconductor behavior. These properties attributed the use of zirconia in a number of industrially important chemical reactions (Fig. 1) [8].

Various zirconia-based catalysts were reported for the synthesis of coumarin through Pechmann reaction. Coumarin belongs to a class of flavonoids and a type of benzo-2-pyrone, which is a plant secondary metabolite isolated from natural plants and some microorganisms. For instance, the antibiotic novobiocin, coumermycin A_1_, and chlorobiocin were isolated from microorganisms [1, 2]. Coumarin acts as a safeguard against viral, bacterial, and fungal attacks, wounds, and stress by a process called phytoalexins [3, 4]. The potential biological applications of coumarin were reported as platelet aggregation inhibition, antibacterial, anticancer, and antioxidant [7, 8]. Coumarin and its derivatives are widely used in synthetic, pharmaceutical, and agrochemicals industries and also used as optical brightening agents, insecticidal, additive in perfumes, and cosmetics [5, 6]. Coumarine serves as an intermediate in the synthesis of several organic reactions, i.e., furocoumarins, chromenes, coumarones, and 2-acylresorcinols [9]. Calanolides, a polycyclic coumarin, exhibited potent anti-HIV (NNRTI) activity and was isolated from genus *Calophyllum* [10].

The bioavailability of coumarin is sessional and environment dependent, so its production is variable at large scale from the natural resources. However, the remarkable application of coumarin and its derivatives needs it at large scale in medicinal, pharmaceutical, synthetic, and several other industries. Coumarin has been prepared through various strategies such as Perkin [11], Pechmann [12], Reformatsky [11], Knoevenagel [13], Wittig reactions [14], and flash vacuum hydrolysis [15]. Among all these reactions, Pechmann reaction was found as the most effective for this synthesis. Formerly, concentrated H_2_SO_4_ was employed for the synthesis of coumarin in Pechmann reaction. Several inorganic reagent and Lewis acid such as P_2_O_5_, FeCl_3_, ZnCl_4_, POCl_3_, AlCl_3_, PPA, HCl, phosphoric acid, trifluoroacetic acid, and montmorillonite clays were used for the synthesis of this scaffold [9]. A number of other catalysts were also successfully reported in the literature for this condensation reaction, i.e., Nafion-H, W/ZrO_2_ solid acid, zeolite H-BEA, montmorillonite clay, ionic liquids, and Amberlyst-15 [10].

The Pechmann reaction is an acid-catalyzed reaction that proceeds through three main steps. The first step is transesterification, which involved an exchange between phenol and *β*-ketoester followed by intramolecular hydroxyl alkylation in the second step and elimination of a water molecule in the third step as depicted in Fig. 8 [10, 16]. Therefore, the yield of an acid catalyzes reactions depends on the acidic strength of the catalyst [17].

A large number of reactions are preceded in the presence of hazardous catalysts that deteriorate the climatic condition. Therefore, an environmentally benign alternative catalyst is needed for those reactions that are catalyzed by expensive ionic liquid, hazardous acid, and toxic catalyst [18–20]. This need can be fulfilled by the use of a catalyst that not only furnishes the required targets but also is eco-friendly. At present, zirconia got much attention as a solid acid catalyst in terms of their acidic strength, recyclability, and environmental benignity.

Based on the acidic strength of zirconia, we carried out Pechmann reaction with different zirconia-based catalyst (ZrO_2_-TiO_2_, ZrO_2_-ZnO, ZrO_2_/cellulose) that acts as a solid acid catalyst. The reaction was carried out under the solvent-free condition as well as in ethanol and toluene solvent. The kinetics of the reaction was studied for the first time for this reaction. The structures of the mentioned catalyst were determined by field emission electron microscope (FESEM), energy dispersive X-rays spectrometry (EDS), X-ray diffraction (XRD), and Fourier transform infrared spectroscopy (FT-IR). This method has several advantages such as simplicity of the reaction, solvent-free condition, room temperature, inexpensive starting material, no side product, high yield, high reaction rate, and no toxic waste material.

## Experimental

### Materials

Reagents such as a salt of zinc and zirconium nitrates, NaOH, cellulose acetate, and TiO_2_ were purchased from Sigma-Aldrich. Departmental Millipore-Q water purification assembly was used for deionized water. Ethyl acetoacetate and phenols (resorcinol and catechol) were taken from Koch-Light Laboratories Ltd.

### Synthesis of Nanomaterial

#### Synthesis of ZrO_2_-TiO_2_

The nanoparticle ZrO_2_-TiO_2_ was synthesized according to our previous reports [21–24]. The commercially available TiO_2_ was treated with the aqueous solution of Zr(NO_3_)_2_. The solution was basified with 0.1 M NaOH solution till the pH reached 9. The reactants were stirred vigorously for 24 h and the supernatant was removed by centrifugation to isolate the precipitate of ZrO_2_-TiO_2_. The procedure of centrifugation is repeated for three times by washing with ethanol. Finally, the resultant precipitate was washed with 1:1 water/ethanol solvent mixture for several times and dried at 50 °C for 24 h in an oven.

#### Synthesis of ZrO_2_-ZnO

The ZrO_2_-ZnO flowers were synthesized by the same method as employed for ZrO_2_-TiO_2_. An equimolar mixture of salts of Zn and Zr nitrates were mixed together and increased the pH of the solution above 11 by dropwise addition of 0.1 M NaOH solution. The resultant basified solution was kept on stirring for 24 h at 50 °C. After stirring, the precipitate was washed with ethanol and centrifuged to remove the supernatant solution. The resultant precipitate was finally washed with H_2_O:C_2_H_5_OH (1:1) mixture and then dried in an oven at 50 °C for 24 h.

#### Synthesis of ZrO_2_/Cellulose

ZrO_2_ nanoparticle was grown on the surface of cellulose by adding 1:1 mixture of cellulose and Zr(NO_3_)_2_ [25]. The solution mixture was basified with 0.1 M NaOH solution in order to facilitate the formation of the nanoparticle. Finally, the precipitate was centrifuged and washed with 1:1 H_2_O:C_2_H_5_OH mixture and dried at 50 °C in the oven for 24 h.

### Characterization of Nanomaterials

The nanomaterials (ZrO_2_-TiO_2_, ZrO_2_-ZnO_,_ and ZrO_2_/cellulose) were extensively studied through spectroscopic techniques. FESEM, JEOL (JSM-7600F, Japan), was used to find the morphology and average size of the nanomaterials. EDS oxford-EDS system was employed to investigate the elemental composition of the nanomaterials. The structures of nanomaterials were further analyzed by ARL X’TRA X-ray Diffractometer. The functional group in nanomaterial was characterized by FT-IR (Thermo scientific), while kinetics of the reactions were studied by UV/Visible spectrophotometer (Thermo scientific), and the product was identified through melting point (Buchi).

## Results and Discussion

### Structure Characterization of Nanoparticles

**Fig. 2:**
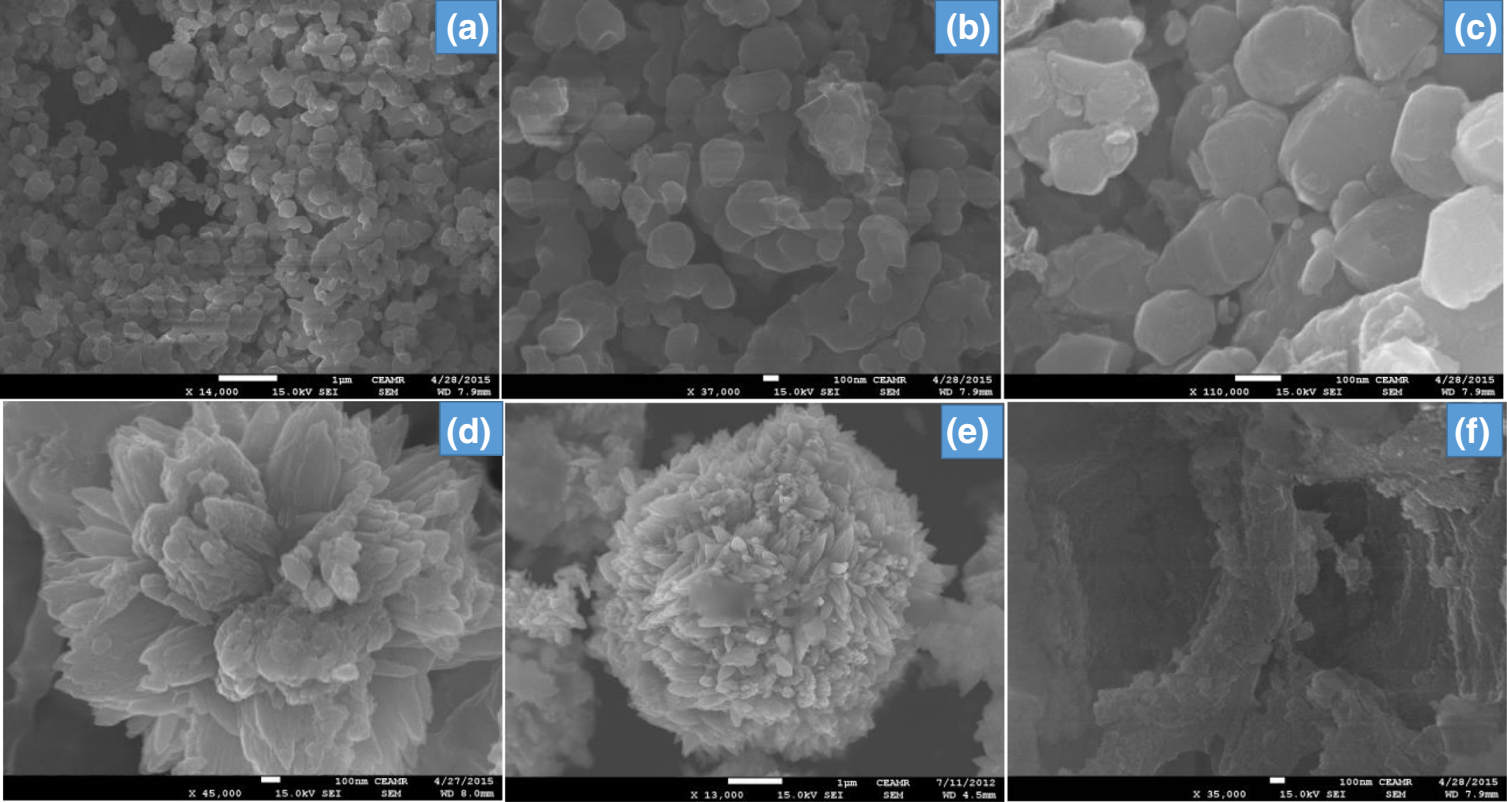
FESEM images of ZrO_2_-TiO_2_ (**a**-**c**), ZrO_2_-ZnO (**d**, **e**), and ZrO_2_/cellulose (**f**)

The morphology of ZrO_2_-TiO_2_, ZrO_2_-ZnO, and ZrO_2_/cellulose was largely characterized by FESEM. ZrO_2_-TiO_2_ was grown in the form of particles (Fig. 2a–2c) while the ZrO_2_-ZnO was grown in flower shape (Fig. 2d, 2e). ZrO_2_-ZnO was basically grown in the form of nanoparticles with an average size of 25–30 nm which aggregate to make a flower-shaped structure. In the case of ZrO_2_/cellulose, ZrO_2_ was grown in the form of particles on the surface of cellulose as shown in Fig. 2f.

**Fig. 3:**
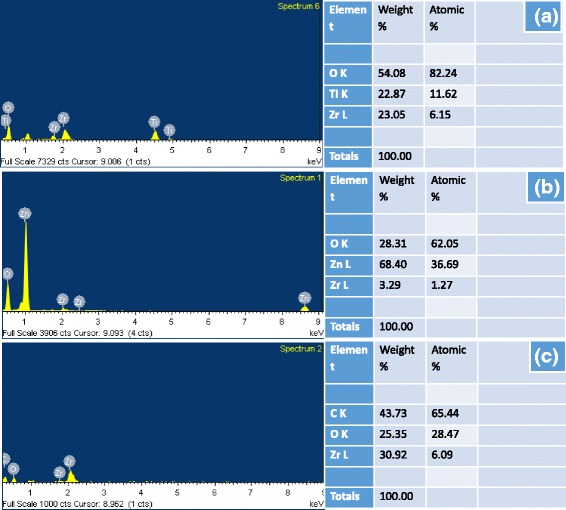
EDS spectrum and elemental composition of ZrO_2_-TiO_2_ (**a**), ZrO_2_-ZnO (**b**), and ZrO_2_/cellulose (**c**)

The elemental composition of ZrO_2_-TiO_2_, ZrO_2_-ZnO, and ZrO_2_/cellulose were performed by EDS spectroscopy as indicated in Fig. 3a–3c. The EDS spectrum of ZrO_2_-TiO_2_ nanoparticle revealed peaks for O, Ti, and Zr elements, in which the weight of Ti, Zr, and O was 22, 23, and 54 %, respectively, as shown in Fig. 3a. Similarly, ZrO_2_-ZnO exhibited peaks for O, Zn, and Zr element, having Zn, Zr, and O element in 68, 3, and 28 % by weight as indicated in Fig. 3b. The ZrO_2_/cellulose displayed peaks for C, Zr, and O element which are 43, 25, and 30 % by weight respectively as shown in Fig. 3c.

**Fig. 4:**
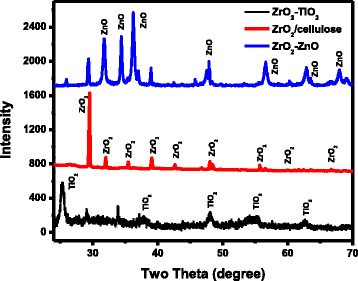
XRD spectrum of ZrO_2_-TiO_2_, ZrO_2_-ZnO, and ZrO_2_/cellulose

Figure 4 shows XRD spectrum of ZrO_2_-TiO_2_, ZrO_2_-ZnO, and ZrO_2_/cellulose. ZrO_2_/cellulose nanomaterial has ZrO_2_ in monoclinic crystalline phase [25]. TiO_2_/ZrO_2_ and ZrO_2_-ZnO nanomaterials contain both TiO_2_ and ZnO phases along with ZrO_2_ phase, respectively. By comparing the intensities of two phases in ZrO_2_-TiO_2_ and ZrO_2_-ZnO nanomaterials, it can be seen that TiO_2_ and ZnO are the major components in ZrO_2_-TiO_2_ and ZrO_2_-ZnO, respectively.

**Fig. 5:**
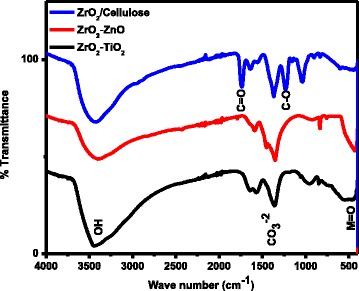
FT-IR analysis of ZrO_2_-TiO_2_, ZrO_2_-ZnO, and ZrO_2_/cellulose

The functional groups in the nanomaterials (ZrO_2_-TiO_2_, ZrO_2_-ZnO, and ZrO_2_/cellulose) were explored by FT-IR spectrophotometer as indicated in Fig. 5. The FT-IR spectrum of all the nanomaterials exhibited a peak at around 500 cm^−1^ indicating stretching vibration for M = O in ZrO_2_-ZnO, ZrO_2_-TiO_2_, and ZrO_2_/cellulose. The sharp signal for carbonate anions appeared in the FT-IR spectrum of ZrO_2_-TiO_2_ and ZrO_2_-ZnO at 1362 and 1343 cm^−1^, respectively. The absorption peak at 3450 cm^−1^ confirmed the presence of OH stretching vibration. The peak for OH stretching vibration is the most prominent in ZrO_2_-TiO_2_ and ZrO_2_/cellulose while it is very weak in ZrO_2_-ZnO. A prominent peak appeared at 1739 cm^−1^ suggesting the presence of carbonyl group and the peak at 1224 cm^−1^ indicating the C–O bond in ZrO_2_/cellulose_._ The FT-IR data suggested that ZrO_2_-ZnO and ZrO_2_-TiO_2_ are metal oxides while in the case of ZrO_2_/cellulose, the ZrO_2_ is supported by cellulose [10, 15, 25, 26].

**Fig. 6:**
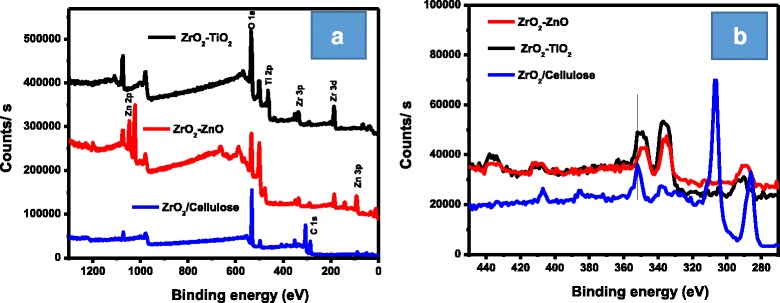
XPS data of ZrO_2_-TiO_2_ (**a**), ZnO-ZrO_2_ (**b**), and ZrO_2_/cellulose

By the bombardment of X-ray, the number of electrons ejected from the surface of the sample was determined by X-ray photoelectron spectroscopy (XPS) as shown in Fig. 6a–6c. ZrO_2_-TiO_2_ exhibited peaks for oxygen, titanium, and zirconium (O 1s, Ti 2p, Zr 3P, Zr 3d, and Zr 4P) while ZrO_2_-ZnO showed peaks for zinc, zirconium, and oxygen (O 1s, Zn 2P, Zn 3P, Zr 3P, Zr 3d and Zr 4p). Similarly, ZrO_2_/cellulose exhibited peaks for O 1s, C 1s, Zr 3P, and Zr 4P. Ti 2P, Zn 2P, and Zn 3P appeared in the XPS spectra at binding energies of 500.0, 1076, and 91.9 eV, respectively, as depicted in Fig. 6a. Zr 4P, Zr 3d, and Zr 3P appeared in the XPS spectra having binding energies of 350, 329, 37.9, and 1072.3 eV. Similarly, O 1s and C 1s were displayed at 535 and 185.0 eV in the XPS spectra as shown in Fig. 6a. The expanded XPS detailed spectra for all the materials are shown Fig. 6b. One can obviously see in these figures that Zr 3p peaks are shifted towards lower binding energies in both ZrO_2_-TiO_2_ and ZrO_2_-ZnO as compared to Zr 3p peak position in ZrO_2_/cellulose. Similar shift behavior has been reported [27] and can be attributed to the formation of ZrO_2_-TiO_2_ and ZrO_2_-ZnO binary oxides.

### General Description for the Synthesis of Coumarin

The reaction was carried out between resorcinol and ethyl acetoacetate (1:2) by using 50 mg of the catalyst ZrO_2_-TiO_2_ in three-neck round-bottom flask in solvent-free condition at room temperature. The resultant product was formed without side product with a m.p. of 184–187 °C. The diagrammatic view of the reaction is depicted in Fig. 1. The reaction was also carried out between resorcinol and ethyl acetoacetate without a catalyst at 80 °C, but no product is formed as shown in Table 1. ZrO_2_-TiO_2_ showed good results as compared to ZrO_2_-ZnO and ZrO_2_/cellulose. The ZrO_2_/cellulose was inactive for this reaction. The reaction was also carried out between catechol and ethyl acetoacetate (1:2) with 50 mg of the catalyst ZrO_2_-TiO_2_ in a solvent-free condition. After 4 h, the reaction between catechol and ethyl acetoacetate gives 55 % yield at 80 °C but failed at room temperature as shown in Table 1. The reaction gives good yield with electron donating group such as resorcinol while failed with electron withdrawing group *O*-nitrophenol as shown in Table 1. Due to the strongest catalytic performance of ZrO_2_-TiO_2_ with resorcinol and ethyl acetoacetate, we further select this catalyst for the detailed study of this reaction. The reaction between resorcinol and ethyl acetoacetate (1:2) with 50 mg of the catalyst ZrO_2_-TiO_2_ was studied in a polar solvent (ethanol) and non-polar solvent (toluene) by varying the temperature condition (Table 2). The use of solvent-free condition is a better way while using a heterogeneous catalyst. Prior to the use of a catalyst, the reaction was carried out between resorcinol and ethyl acetoacetate in the absence of a catalyst in a solvent-free condition, toluene, and ethanol, but no product is formed. This confirms that solvent or temperature have no role; only catalyst played a central role in this reaction.

**Table 1:** The reaction in solvent-free condition at room temperature

Entry	Reactant	Catalyst	Temperature (°C)	Time (min.)	%Yield	M.P.
1	Resarcinol + ethylacetoacetate	ZrO_2_-TiO_2_	R.T.	180	97	184–187
2	Resarcinol + ethylacetoacetate	ZrO_2_-ZnO	R.T.	240	63	184–187
3	Resarcinol + ethylacetoacetate	ZrO_2_/cellulose	R.T.	180	N.R.	–
4	Catechole + ethylacetoacetate	ZrO_2_-TiO_2_	80	240 min	55	
5	*O*-nitrophenol + ethylacetoacetate	ZrO_2_-TiO_2_	80	240	N.R.	
6	Resarcinol + ethylacetoacetate	Without catalyst	80	240	N.R.	–

*N.R.* no reaction, *M.P.* melting point

**Table 2:** Comparison of the present work with literature data

Entry	Catalyst	Time (min.)	Temperature (°C)	Solvent	Yield	References
1	Zeolite BEA	240	130	PhNO_2_	63	[29]
2	PFPAT	180	110	Toluene	90	[30]
3	MFRH	50	80	S.F.	65	[30]
4	Nanoreactors	60	130	S.F.	30	[30]
5	CMK-5-SO3H	20	130	S.F.	95	[7]
6	CMK-5	60	130	S.F.	10	[7]
8	ZrO_2_-TiO_2_	180	Room temperature	S.F.	97	This work
9	ZrO_2_-TiO_2_	110	60	Toluene	95	This work
10	ZrO_2_-TiO_2_	150	60	Ethanol	92	This work

*PhNO*_*2*_ nitrophenol, *S.F.* solvent-free condition

### Temperature Effect

The temperature effect was observed on the reaction ZrO_2_-TiO_2_ (50 mg) in the presence of toluene and ethanol. It was observed that increasing the temperature will decrease the time for reaction completion as indicated in Tables 3 and 4.

**Table 3:** Effect of temperature in toluene solvent

Entry	Reactant	Catalyst	Temperature (°C)	%Yield	Time (min.)
1	Resarcinol + ethylacetoacetate	ZrO_2_-TiO_2_	100	95	20
2	Resarcinol + ethylacetoacetate	ZrO_2_-TiO_2_	80	95	35
3	Resarcinol + ethylacetoacetate	ZrO_2_-TiO_2_	60	95	110
4	Resarcinol + ethylacetoacetate	ZrO_2_-TiO_2_	45	83	130
5	Resarcinol + ethylacetoacetate	ZrO_2_-ZnO	100	70	30
6	Resarcinol + ethylacetoacetate	ZrO_2_-ZnO	80	63	50
7	Resarcinol + ethylacetoacetate	ZrO_2_-ZnO	60	55	140
8	Resarcinol + ethylacetoacetate	ZrO_2_-ZnO	45	40	170
9	Resarcinol + ethylacetoacetate	Without catalyst	80	N.R.	240

*N.R.* no reaction

**Table 4:** Effect of temperature in an ethanol solvent

Entry	Reactant	Catalyst	Temperature (°C)	%Yield	Time (min.)
1	Resarcinol + ethylacetoacetate	ZrO_2_-TiO_2_	100	97	20
2	Resarcinol + ethylacetoacetate	ZrO_2_-TiO_2_	80	95	50
3	Resarcinol + ethylacetoacetate	ZrO_2_-TiO_2_	60	92	150
4	Resarcinol + ethylacetoacetate	ZrO_2_-TiO_2_	45	80	160
5	Resarcinol + ethylacetoacetate	ZrO_2_-ZnO	100	74	40
6	Resarcinol + ethylacetoacetate	ZrO_2_-ZnO	80	67	60
7	Resarcinol + ethylacetoacetate	ZrO_2_-ZnO	60	60	120
8	Resarcinol + ethylacetoacetate	ZrO_2_-ZnO	45	43	150
9	Resarcinol + ethylacetoacetate	Without catalyst	80	N.R.	240

*N.R.* no reaction

Ethyl acetoacetate and resorcinol (1:2) was used as starting materials for the synthesis of coumarin along with 50 mg of the catalyst.

### UV/Visible Data

**Fig. 7:**
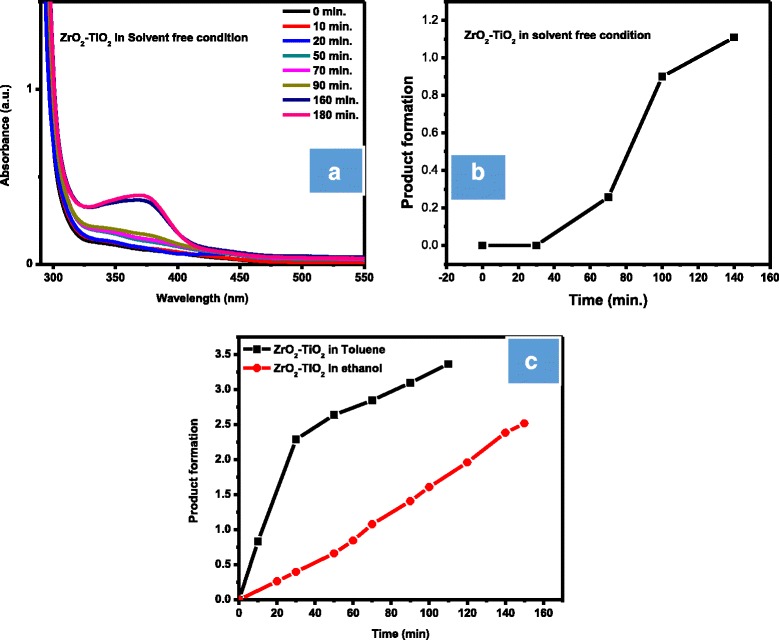
**a** UV/Vis data of ZrO_2_-TiO_2_ in solvent-free condition at room temperature. **b** Kinetics of ZrO_2_-TiO_2_ in solvent-free condition. **c** Kinetics of ZrO_2_-TiO_2_ in toluene and ethanol solvent at 60 °C

The increase in product concentration was monitored gradually by taking the UV/Visible spectra periodically. A bathochromic shift was observed for the product, due to an increased conjugation as compared to the reactant. However, the product showed a different bathochromic shift in ethanol and toluene solvent. The bathochromic shift (increase in wavelength) was observed in ethanol at 372 nm while the same product appeared at 317 nm in toluene. In the presence of non-polar solvent (toluene), polar molecule showed hypsochromic shift due to n-π^∗^ transition because it stabilizes the ground state more as compared to the excited state; therefore, a high amount of energy is required to promote an electron from the highest occupied molecular orbital (HOMO) of non-bonding orbital to the lowest unoccupied molecular orbital (LUMO) of antibonding π^∗^ orbital, and so the wavelength is decreased. However, polar solvent (ethanol) forms hydrogen bonding to the excited state of the product (coumarin), which stabilizes the transition state of the product more as compared to the ground state. Therefore, less amount of energy is required to promote an electron from HOMO of non-bonding orbital to the LUMO of the antibonding π^∗^ orbital and thus increasing the wavelength as shown in Fig. 7a.

### Kinetics of the Reaction

The kinetics was studied in solvent-free condition, ethanol, and toluene in the presence of ZrO_2_-TiO_2_ catalyst. The rate of reaction in solvent-free condition at room temperature was 1.7 × 10^−3^ g mol^−1^ min^−1^, while at 60 °C the rate of reaction in ethanol is 1.7 × 10^−2^ g mol^−1^ min^−1^ and toluene 5.6 × 10^−3^ g mol^−1^ min^−1^ as shown in Fig. 7a–7c.

### Mechanism of the Reaction

**Fig. 8:**
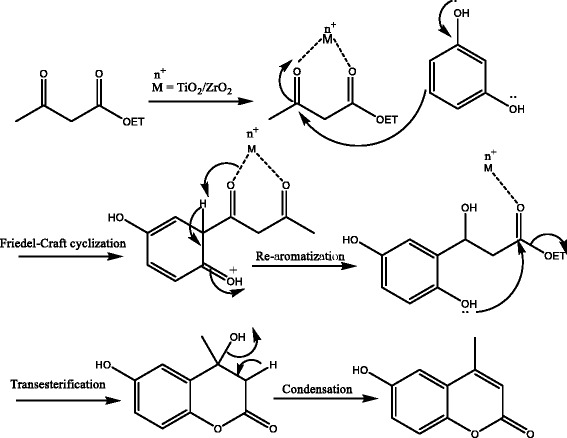
Tentative mechanism for the synthesis of coumarin

Several mechanisms were put forward for the synthesis of coumarin. In the whole scenario, one C–O and one C–C bond are generated by the reaction of phenol with *β*-ketoester [28]. During C–C bond formation, the metal in the nanocatalyst chelates with *β*-ketoester, followed by Friedel-Craft cyclization in which the π-electron of the benzene ring of phenol attacks the carbonyl carbon of *β*-ketoester to form an unstable anti-aromatic species (4n electron system). This highly unstable anti-aromatic species restore its aromaticity (4n + 2π electron system) by losing hydrogen atom. Transesterification occurred in the next step followed by condensation to form C–O bond as depicted in Fig. 8.

## Conclusions

In the present study, zirconia-based catalysts (ZrO_2_-TiO_2,_ ZrO_2_-ZnO, ZrO_2_/cellulose) were synthesized for the one-pot synthesis of coumarin. The ZrO_2_-TiO_2_ showed strongest catalytic performance for this reaction as compared to ZrO_2_-ZnO. At room temperature, the rate of reaction in solvent-free condition is 1.7 × 10^−3^ g mol^−1^ min^−1^. However, at 60 °C, the rate of reaction in ethanol is 1.7 × 10^−2^ and toluene 5.6 × 10^−3^ g mol^−1^ min^−1^. The rate of reaction was increased by increasing the temperature of the reaction. The bathochromic shifts was observed in the UV/Visible spectrum of the ethanol. The product appeared at *λ*_max_ 372 nm in the presence of the ethanol as it stabilized the excited state of the polar molecule (coumarin). Similarly, the product appeared at *λ*_max_ 317 nm in toluene solvent as it stabilizes the ground state of the polar molecule (coumarin).
